# Effects of high-intensity interval cross training and classical training on physical and sprint performance in male junior sprinters

**DOI:** 10.1371/journal.pone.0354592

**Published:** 2026-08-03

**Authors:** Zhenyu Zhang, Chenghao Liu, Tao Liu, Yun Xie

**Affiliations:** 1 College of Physical Education, Tianjin University of Sport, Tianjin, China; 2 College of Exercise, Tianjin University of Sport, Tianjin, China; SPRINT - Sport Physical Activity and Health Research & Innovation Center, PORTUGAL

## Abstract

High-Intensity Interval Cross Training (HIICT) combines sprinting, plyometric, and functional strength exercises, but its effects compared with classical sprint training in male junior sprinters remain unclear. This study examined the effects of HIICT and Classical Training (CT) on physical and sprint performance in male junior sprinters. Sixty-four male junior sprinters were randomly assigned to an HIICT group or a CT group for an 8-week intervention. Both groups completed similar overall internal training loads. Assessments before and after the intervention included sprint performance, jump performance, ground contact time, strength-related outcomes, anaerobic power, and body composition. Internal load was monitored using session rating of perceived exertion. Training load was comparable between groups throughout the intervention. Compared with CT, HIICT showed more favorable changes in 100-m sprint performance, first-60-m split time, ground contact time, countermovement jump performance, and relative peak power. Squat jump performance improved in both groups, with a greater tendency toward improvement following HIICT. Changes in body composition and several strength-related outcomes were broadly similar between groups, whereas power-clean performance showed a more favorable response in the HIICT group. These findings suggest that, under comparable internal training loads, HIICT may provide additional benefits for sprint-related explosive qualities and acceleration-related performance in male junior sprinters. From a practical perspective, HIICT may be considered as a supplementary training option within junior sprint training programs, particularly when the aim is to target sprint-specific neuromuscular qualities. However, given the specific sample and methodological limitations, these findings should be interpreted cautiously and require confirmation in future studies.

## Introduction

High-intensity interval training (HIIT) is commonly applied in track and field, especially within sprint-hurdle [1] and middle-distance preparation [2], to develop anaerobic power and to improve aerobic recovery between repeated high-intensity efforts [3]; however, its programming and primary adaptations differ from those targeted in long-distance endurance events [4]. Traditional HIIT protocols are typically characterized by repeated bouts of high-intensity exercise interspersed with brief recovery periods, allowing athletes to achieve substantial physiological stress within a relatively short training duration [3]. In contrast to HIIT, which usually relies on repeated high-intensity bouts within a single or dominant exercise mode, HIICT combines multiple exercise modalities, such as sprinting, jumping, and functional strength exercises, within an interval-based structure. Previous HIIT studies have demonstrated improvements in maximal oxygen uptake (VO_2_max) and maximal accumulated oxygen deficit (MAOD), both of which are relevant to high-intensity exercise capacity [5,6]. However, these adaptations should be distinguished from direct evidence for HIICT effects in junior sprinters. Cross-training originated from early athletic training practices that combined endurance and explosive strength exercises [7]. Cross-training integrates diverse exercise modalities into a single training program to stimulate multiple physiological systems [7,8], fostering comprehensive athletic development [9]. While traditional training programs, such as Classical Training (CT), often focus on developing specific athletic skills (e.g., running, swimming, or strength), cross-training aims to develop multiple skills concurrently, reducing the risk of overtraining and injuries.

High-Intensity Interval Cross-Training (HIICT) has been developed as an innovative approach incorporating various exercise modalities to overcome these limitations. By combining running, jumping, squatting, and other activities, HIICT may provide diverse stimuli for anaerobic metabolism and athletic performance while reducing the fatigue and monotony often associated with single-mode training [4]. This approach may improve training sustainability and applicability in young athletes. HIICT may also be relevant to junior athletes because it combines high training intensity with broader physical development [10]. Despite its theoretical foundation and some reported benefits, systematic research on the effects of HIICT on the specialized abilities of junior sprinters remains limited.

Despite the many training methods applied to youth sprint training, the extent to which different high-intensity and combined training approaches transfer to sprint-specific performance remains unclear. Existing evidence should be interpreted according to the populations and outcomes examined in each study. For example, high-intensity intermittent cross-training has been reported to improve maximal oxygen uptake, but this evidence mainly supports cardiorespiratory adaptations rather than direct sprint-performance benefits in junior sprinters [4]. Evidence from youth training studies is more directly relevant to the present context. A systematic review by Behm et al. showed that strength and power training can improve muscle strength, power, and speed-related outcomes in youth, although the magnitude of adaptation depends on the training modality and outcome assessed [11]. In adolescent sprinters, functional, traditional, and combined training approaches have also been compared for sprint-related performance, suggesting that different training models may produce distinct adaptations in acceleration and sprint outcomes [12]. Therefore, the remaining gap is not whether high-intensity or combined training can improve general physical fitness, but whether an interval-based, multi-modal HIICT program provides additional sprint-specific benefits over classical sprint training in male junior sprinters.

Moreover, dedicated studies on high-intensity intermittent cross-training (HIICT) in junior sprinting remain scarce, leaving a clear research gap. On one hand, much of the evidence for HIICT derives from adults or youths in team sports rather than sprint-specialized junior athletes. Whether such findings translate directly to male junior sprinters older than 15 years is still unknown. On the other hand, in this population, the effects of integrating sprint- and jump-based functional drills with conventional strength training have not been systematically characterized. Although available evidence suggests that hybrid programs may confer broader fitness benefits [13,14], evidence evaluating this specific HIICT protocol and its transfer to sprint-specific performance outcomes in male junior sprinters older than 15 years remains limited. Accordingly, controversies persist over the superiority of HIICT versus traditional routines, the possibility of synergistic benefits, and their longer-term impact in this population, all calling for further clarification. We therefore examined an eight-week HIICT intervention in male junior sprinters older than 15 years, assessing body composition, sprint outputs, and physiological performance indicators. Classical training (CT) served as the control, aiming to address the current knowledge gap and advance the practical application of innovative sprint training regimens tailored for junior sprinters. We hypothesized that, under comparable internal training loads, HIICT would produce greater improvements than CT in sprint-specific outcomes, particularly 100-m sprint time, first-60-m split time, ground contact time, jump performance, and relative peak power, in male junior sprinters older than 15 years.

## Materials and methods

### Participants

Sample size was determined a priori using G*Power (version 3.1) for an F-test (ANOVA: repeated measures, within–between interaction), assuming an effect size of f = 0.20, α = 0.05, statistical power (1 − β) = 0.90, two groups, two measurements, a correlation among repeated measures of 0.60, and a nonsphericity correction of ε = 1.00. The minimum required sample size was 56 participants (28 per group). Finally, 70 volunteered for screening, 64 eligible athletes were included. At baseline, participants were 17.54 ± 1.19 years old, had 2.34 ± 0.56 years of training experience, were 1.79 ± 0.04 m in height, had a body mass of 69.77 ± 2.34 kg, and had a 100-m sprint time of 11.46 ± 0.25 s. The inclusion criteria were selected to ensure that participants represented male junior sprinters with sufficient training background to safely complete the intervention, while minimizing the influence of prior HIICT exposure and recent injury. Participants were eligible if they met the following criteria: (1) they were older than 15 years and had obtained consent from their school and legal guardians; (2) they had no prior experience with HIICT; (3) they had completed at least six months of systematic sprint training before the experiment; and (4) they had no exercise-related illness and no sports injury within the previous three months.

This study was conducted in accordance with the Declaration of Helsinki and was approved by Tianjin University of Sport (Approval No: TJUS-2025–004) (Table 6 summarize baseline and post-intervention outcomes for the HIICT and CT groups (S1 File). Participants were recruited from an athletics club between February 1 and February 15, 2025. After screening, 64 eligible athletes were included in the study. All participants were informed of the study procedures, potential benefits, and possible risks before participation. Written informed consent was obtained from all participants. For participants under 18 years of age, written consent was also obtained from one legal guardian.

During testing and training sessions, all participants were supervised by qualified coaches and research staff. Potential risks were similar to those encountered in routine sprint training, including transient fatigue, muscle soreness, cardiorespiratory discomfort, and minor sports injuries. If marked discomfort occurred, the session was stopped or modified immediately, and on-site management or referral for medical evaluation was provided when necessary.

### Randomization, allocation concealment, and blinding

After baseline assessments, participants were assigned unique study identification codes for data management and blinded outcome assessment. Participants were then randomly allocated to the HIICT or CT group in a 1:1 ratio using a simple randomization sequence generated by Author I with Random.org. The allocation sequence was stored securely and concealed until all baseline testing had been completed. Group assignments were then disclosed only to the researchers responsible for implementing the training programs.

**Fig 1:**
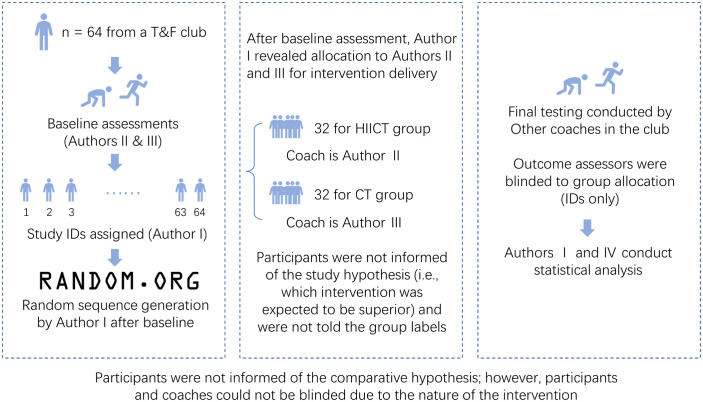
Randomization, allocation, and blinding procedures of the trial. After baseline assessment and assignment of study identification numbers, 64 participants were randomly allocated to the HIICT or CT group. The outcome assessors were blinded to group allocation, and the statistical analyses were conducted using coded group labels. Participants and intervention coaches could not be blinded because of the nature of the interventions. HIICT, high-intensity interval cross training; CT, classical training; T&F, track and field.

Participants and coaches could not be blinded to the intervention type because the training protocols were visibly different. Therefore, participants may have been able to infer their group allocation during the intervention. To reduce expectation bias, participants were not informed about the study hypothesis or which intervention was expected to be superior. Post-intervention outcome testing was conducted by independent coaches from the same club who did not participate in daily training and were blinded to group allocation. These assessors recorded results using the pre-assigned study identification codes only. Statistical analyses were performed by Authors I and IV using coded group labels (A/B), and the group codes were not disclosed until the main analyses had been completed (Fig 1).

### Procedure

An 8-week intervention training program was implemented for all athletes. Testing was conducted both at the beginning and the end of the training period, spanning five days each time. The testing protocol was as follows (Table 1):

**Table 1:** Testing Schedule. ^: Test three times and record the best result.

Days	Morning	Afternoon
1	Height; Weight; CMJ^; SJ^	
2	Power clean^	Snatch^
3		100m Sprint
4	Squat^	Bench press ^
5	200m Sprint	Anaerobic power

Day 1: Measurement of height and weight, countermovement jump (CMJ), and static squat jump (SJ). Day 2: Testing of 10RM in the power clean (morning) and 10RM in the snatch (afternoon). Day 3: Testing of 100m sprint. Day 4: Testing of 10RM in the squat (morning) and 10RM in the bench press (afternoon). Day 5: Testing of 200m sprint (morning) and anaerobic power (afternoon). This sequence was arranged to reduce fatigue carryover between tests with high neuromuscular demands. Sprint tests were separated from lower-body strength tests, and when two tests were performed on the same day, they were scheduled in separate morning and afternoon sessions with sufficient recovery.

To promote safe participation and reduce injury risk in adolescent athletes, maximal strength was not assessed using 1RM testing. Instead, 10RM tests were used as practical field measures of exercise-specific strength or strength-endurance capacity in the squat, bench press, power clean, and snatch. These outcomes were included because sprint performance is partly related to the ability to repeatedly produce high force during acceleration and high-speed running. However, the 10RM results should not be interpreted as direct measures of muscular power, lower-limb power, or metabolic energy-system contribution.

A full-frame squat rack was used for the squat test, a flat bench press rack for the bench press, and a weightlifting platform for the power clean and snatch tests. A standard men’s 20 kg barbell and 45 cm Olympic weightlifting plates were used for all strength tests. Before testing, all athletes estimated their 10RM based on their previous training experience and gradually warmed up to an approximate 10RM load. During the formal test, each athlete lifted their estimated load for 10 repetitions. If successful, the weight was increased in 2.5 kg increments until the athlete could no longer complete 10 repetitions. The maximum weight successfully lifted for 10 repetitions was recorded as the athlete’s 10RM [15].

Jump tests were recorded using a single iPhone 15 Pro Max positioned on the athlete’s right side. The camera was aligned with the calibration line and kept fixed to ensure that the full movement was captured consistently across trials.

Athletes stood in a predefined marked area and, upon receiving the start command, performed a maximal-effort vertical jump. Prior to testing, all athletes received standardized familiarization and technical instructions, including a demonstration, to minimize systematic error associated with the flight-time method. Specifically, athletes were instructed to keep the body upright and to land with the lower limbs extended as much as possible (i.e., avoid exaggerated knee/hip flexion at landing) so that the landing posture would be comparable to the take-off posture. A fixed panoramic recording mode was used throughout the movement until completion. Video footage was subsequently analyzed using Kinovea, from which the number of frames corresponding to the flight phase was extracted. The flight phase was defined as the period from take-off to landing, and the number of flight frames was denoted as Nf. Because the recording frame rate was 240 frames·s^-1^, flight time was calculated as follows:


$$\begin{array}{c} t=\frac{N_{f}}{\text{2}\text{4}\text{0}}=N_{f}\times \text{0}.\text{0}\text{0}\text{4}\text{1}\text{7}\;s \end{array}$$ (1)

Jump height was then calculated using the standard flight-time equation:


$$\begin{array}{c} h=\frac{\text{1}}{\text{8}}gt^{\text{2}} \end{array}$$ (2)

where Nf is the number of flight frames, t is flight time in seconds, h is jump height in meters, and g = 9.81 m·s^-2^. Trials with visibly excessive knee/hip flexion at landing that could artificially prolong flight time were deemed invalid and were repeated. No correction factor was applied. Although the best of three trials was retained for subsequent analyses, within-session reliability across the three attempts was evaluated using ICC(2,1)/ICC(2,3) (two-way random-effects, absolute agreement) and CV%. CMJ showed ICC(2,3) = 0.924 (Pre) and 0.949 (Post) with CV = 3.14–3.02%, while SJ showed ICC(2,3) = 0.919 (Pre) and 0.939 (Post) with CV = 4.45–4.22%.

**Fig 2:**
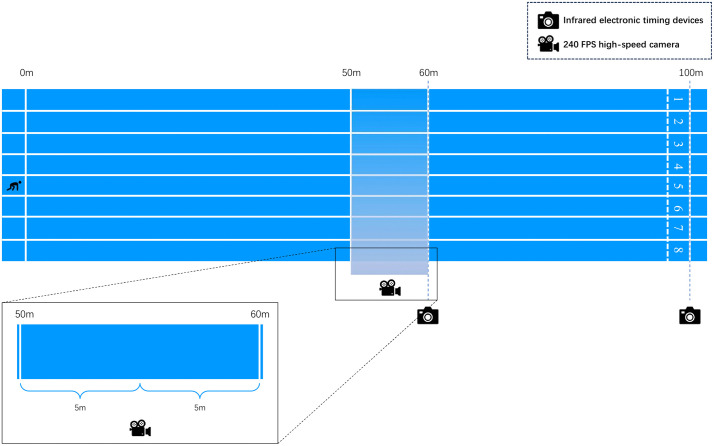
Schematic of the sprint-testing setup and instrumentation (infrared timing gates and 240-fps camera placement) electronic timing devices were positioned at 60 and 100 m to record the first-60-m split and 100-m sprint time, respectively. A 240-frames-per-second high-speed camera was positioned perpendicular to the running direction and centered on the 50–60-m segment to record ground contact time.

The 100-m and 200-m sprint tests were conducted on a standard 400-m track in accordance with the World Athletics rules (2024 edition) and under simulated competition conditions. Infrared electronic timing devices with a timing precision of <0.001 s were used (Hebei Jiechang Sports, China). The starting block (Fairplay, China) automatically detected false starts. Test results were recorded to two decimal places. Ground contact time was assessed only during the 100-m sprint using a video-based analysis method. A single iPhone 15 Pro Max was used for recording (Fig 2), with the camera operator positioned at the highest point of the grandstand and aligned with the extended calibration line connecting the 50 m and 60 m markers, ensuring that both reference points were visible within the camera frame. Previous studies have shown that during the 100-m sprint, athletes typically reach or approach their maximum running velocity between 50 and 80 m, during which ground contact time is relatively short. Therefore, the 50–60 m interval was selected as the experimental zone for ground contact time analysis [16]. Accordingly, GCT in this segment was treated as a field-based running-mechanics variable for the 100-m sprint analysis [17]. This smartphone-based high-speed video approach was used as a field-based method for assessing running mechanics, with previous evidence supporting the validity and reliability of iPhone-based running-mechanics assessment [18]. The recording frame rate was set to 240 frames per second, and panoramic tracking mode was used to ensure that the athlete’s complete passage through the 50–60 m segment was captured. Touch-down was defined as the first frame showing visible foot–ground contact, and toe-off was defined as the last frame showing visible foot–ground contact. For each step, GCT was calculated as the number of contact frames divided by 240. Because one video frame corresponds to approximately 4.17 ms, GCT values were interpreted with this temporal resolution in mind. Video footage was analyzed frame by frame using Kinovea software. The mean GCT across all steps within the 50–60 m segment was used as the final outcome, expressed in seconds and rounded to three decimal places.

Anaerobic power was assessed using the 30-s Wingate Anaerobic Test (WAnT) performed on a mechanically braked cycle ergometer (Monark Ergomedic 894E, Monark Exercise AB, Sweden). Seat height and handlebar position were adjusted individually. Athletes completed a standardized warm-up (5 min of low-resistance pedaling plus two brief 3–5 s accelerations) followed by 3 min of passive rest. At test initiation, athletes accelerated to maximal cadence against minimal resistance, and the braking force was applied immediately using the weight-basket system. The braking force was set at 0.08 kg·kg^-1^ body mass and applied identically at pre- and post-tests. This load is close to the conventional Wingate resistance of 0.075 kg·kg^-1^ body mass and was selected as a practical standardized resistance for junior sprinters with relatively high anaerobic power demands. Because this load was not individually optimized through load–power profiling, it should be interpreted as a pragmatic standardized testing load rather than an individualized optimal resistance. Because this load was not individually optimized through load–power profiling, it should be interpreted as a pragmatic standardized testing load rather than an individualized optimal resistance.

We selected 8% body mass to provide a practical and comparable testing stimulus for junior sprinters while maintaining feasibility and safety in adolescent athletes. This load was not individually optimized through load–power profiling; therefore, it should be interpreted as a pragmatic standardized testing load rather than an individualized optimal resistance. Participants were verbally encouraged to pedal all-out for 30 s. The ATS computes power output from flywheel revolutions and reports power in 5-s epochs; peak power was defined as the highest 5-s power, mean power as the average over the full 30 s, and minimum power as the lowest 5-s power. Fatigue index (30-s power decline) was calculated as (peak power − minimum power)/ peak power × 100. Relative peak power and relative mean power were obtained by dividing absolute values by body mass. [19–22].

Fatigue control was considered when arranging the testing order. Tests with high neuromuscular demands were scheduled on separate days whenever possible. Sprint tests were separated from lower-body strength and power tests. When two tests were performed on the same day, they were conducted in separate morning and afternoon sessions with sufficient recovery. The same testing order was used before and after the intervention to ensure comparability.

### Training plan

**Fig 3:**
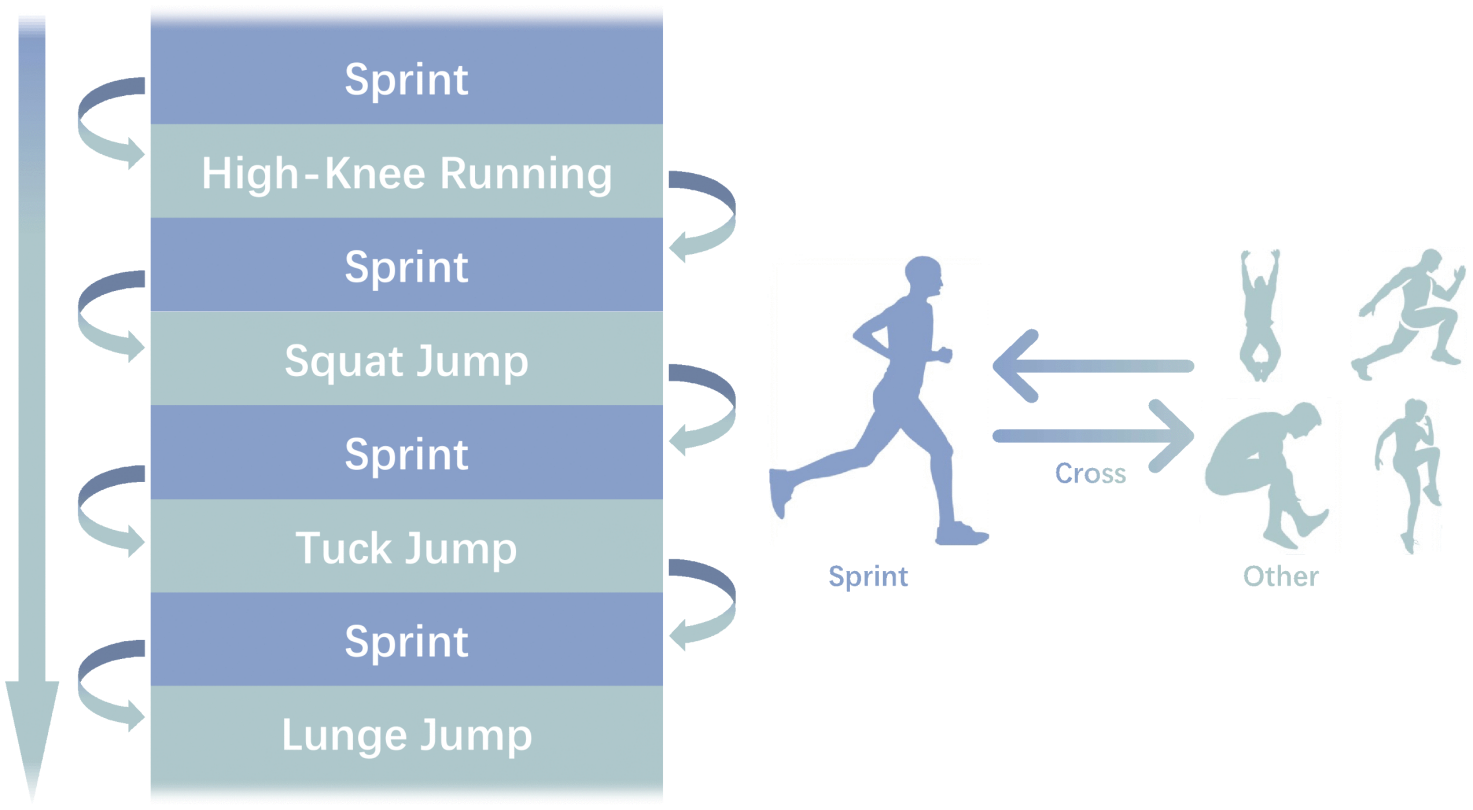
HIICT Training Flowchart. HIICT circuit alternated sprint bouts with high-knee running, squat jumps, tuck jumps, and lunge jumps in the sequence shown. HIICT, high-intensity interval cross training.

For clarity, the term “bout” was used to describe a single 20-s exercise interval, and the term “HIICT set” was used to describe one complete sequence of eight bouts. One HIICT set consisted of eight 20-s high-intensity exercise bouts, with 20 s of passive rest between consecutive bouts. Bout 1, 3, 5, and 7 consisted of sprint training, whereas bout 2, 4, 6, and 8 consisted of high-knee running, squat jumps, tuck jumps, and lunge jumps, respectively (Fig 3; Table 2 and Table 3). Thus, one HIICT set included 160 s of active exercise and 140 s of intra-set rest, for a total duration of 300 s. During each HIICT session, athletes completed two HIICT sets, separated by 12–15 min of rest. Therefore, each HIICT session included 320 s of active exercise and 280 s of intra-set rest, in addition to the 12–15 min between-set rest interval.

**Table 2:** Overview of Training Plans During the Intervention Period.

Days	Training plan
Monday	(1) 30–60-80m sprint × 2 sets (2) Interval Running: 6 × 200 meters, 2-minutes rest intervals
Tuesday	(1) 20-40-60-80 m sprint (2) Interval Running: 2 × 200 meters, 30-seconds rest intervals **Control:** (3) Strength Training:① Fast Lunge Snatch × 3 ② Hang Power Clean × 3 ③ Half Squat + Depth Jump × 3 **Experimental:** (3) HIICT × 2 sets, with 12–15 minutes of rest between sets.
Wednesday	(1) 20-40-60-80 m sprint (2) Interval Running: 8 × 100 meters, 2-minutes rest intervals **Control:** (3) Basic Conditioning Exercises **Experimental:** (3) HIICT × 2 sets, with 12–15 minutes of rest between sets
Thursday	(1) 15–20 minutes of aerobic jogging (2) Hurdle coordination drills and hurdle technique practice (3) 10 movement combination exercises × 3–4 sets, 4-minute rest intervals
Friday	(1) 30-60-80 sprint × 3 sets (2) Interval Running: 2 × 200 meters, 30-seconds rest intervals **Control:** (3) Strength Training: ① Fast Lunge Snatch x 3 × 3 ② Hang Power Clean × 3 × 3 ③ Half Squat + Depth Jump × 3 × 3 ④ Posterior Chain Exercise: 3 × 20 reps **Experimental:** (3) HIICT × 2 sets, with 12–15 minutes of rest between sets
Saturday	(1) 20-40-60-80 m sprint (2) Interval Running: 2 x 200 meters, 30-seconds rest intervals (3) Strength Training: ① Power Clean: 3 × 3 sets ② Snatch: 3 × 3 sets ③ Full Squat or Half Squat: 5 × 3 sets
Sunday	Rest

**Table 3:** Combination Table of HIICT Exercise Training Models.

Method	Intensity	Duration per bout	Bout order
Sprint	85%Vmax	20	1、3、5、7
High-Knee Running	80%Hzmax	20	2
Squat Jump	80%Hzmax	20	4
Tuck Jump	80%Hzmax	20	6
Lunge Jump	80%Hzmax	20	8

### Training load monitoring

Internal training load was monitored for all training sessions using the session rating of perceived exertion (sRPE) method. Athletes reported sRPE approximately 10 min after each session using the Borg CR-10 scale (0 = rest; 10 = maximal). Session duration was recorded in minutes for each training session and defined as the elapsed time of the main training component only (i.e., from the start of the planned workout immediately after the standardized warm-up to the end of the last planned exercise bout), excluding the warm-up and cool-down as well as any extended breaks not planned as part of the session. The internal load of each session was calculated as:


$$\begin{array}{c} Daily\;sRPE\;load\;(AU)=\;sRPE\;\times \;session\;duration\;(min) \end{array}$$ (3)

Weekly internal load was calculated by summing session sRPE-load values within each week. Prior to baseline testing, athletes were familiarized with the CR-10 scale; thereafter, sRPE was collected by the same investigator at a standardized post-session time point throughout the intervention to enhance measurement consistency.

### Training fidelity, adherence, and exposure screening

Training adherence was primarily assessed by (i) session attendance and (ii) protocol completion. Attendance was recorded for every scheduled session using a standardized sign-in log, and all sessions were delivered under direct supervision of the coaching staff. Protocol completion was defined as completing the planned content for each scheduled session. For HIICT sessions, completion required performing all programmed rounds/bouts/sets within the scheduled session. For CT sessions, completion required completing all prescribed exercises and the planned set–rep structure (and load targets, where applicable) as scheduled. As a secondary descriptive check of exposure consistency, weekly internal load (weekly sRPE-load; athlete-week) was monitored and screened for extreme deviations using two a priori criteria: (i) a weekly group-based threshold (group mean ± 2 SD within each week and group) and (ii) an individual-based threshold (each athlete’s mean ± 2 SD across the intervention). These thresholds were used as descriptive screening tools rather than primary indicators of adherence.

### Statistical analysis

All statistical analyses were performed using IBM SPSS Statistics 25 and Microsoft Excel. Before inferential analyses, statistical assumptions were examined. Normality was assessed using the Kolmogorov–Smirnov (K–S) test. Homogeneity of variance between groups was assessed using Levene’s test. Because the within-subject factor had only two levels (pre vs. post), the sphericity assumption was not applicable.

For outcomes that met the assumptions, the primary analysis was a 2 × 2 mixed-design repeated-measures ANOVA, with group (HIICT vs. CT) as the between-subject factor and time (pre vs. post) as the within-subject factor. The Group × Time interaction was treated as the main effect of interest. For outcomes that did not meet the normality assumption, non-parametric tests were used.

Values at each time point are presented as mean ± SD. Changes were calculated as Post − Pre and are presented with 95% confidence intervals. To control Type I error across multiple primary endpoints, 100-m time, first-60-m split, ground contact time, CMJ, SJ, and relative peak power were prespecified as primary outcomes. Holm–Bonferroni correction was applied to the Group × Time interaction p values for these outcomes. All remaining outcomes were considered secondary or exploratory. Partial eta squared (ηp²) was used to quantify the effect size of the Group × Time interaction. The following benchmarks were used for interpretation: small effect, ηp² ≥ 0.01; medium effect, ηp² ≥ 0.06; and large effect, ηp² ≥ 0.14. These benchmarks were treated as heuristic guidelines and were interpreted together with the direction of change, confidence intervals, and practical relevance of each outcome.

## Results

**Fig 4:**
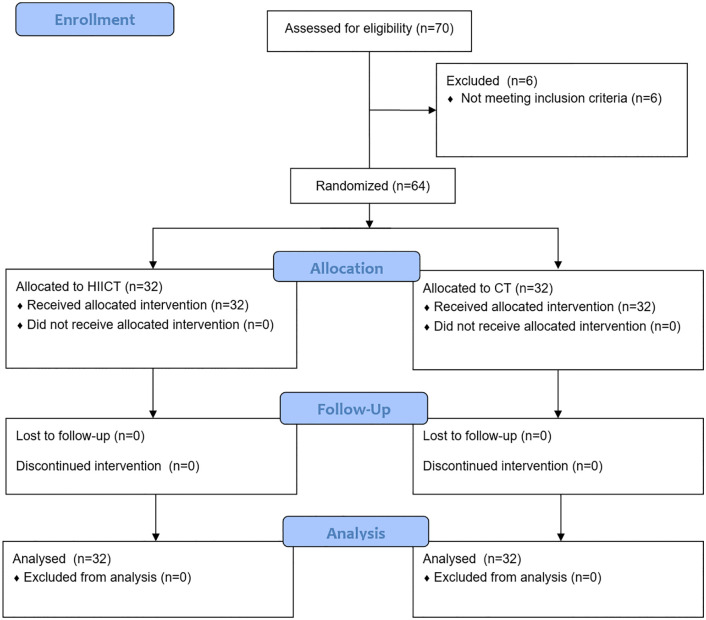
CONSORT flow diagram of participant enrollment, allocation, follow-up, and analysis Of the 70 athletes assessed for eligibility, six did not meet the inclusion criteria. The remaining 64 participants were randomly allocated to the HIICT group or CT group, with 32 participants in each group. All allocated participants completed the intervention and were included in the final analysis. HIICT, high-intensity interval cross training; CT, classical training.

A total of 70 male junior sprinters volunteered for screening at a track-and-field club. Six individuals were excluded before randomization because they did not meet the inclusion criteria. Therefore, 64 eligible participants completed baseline assessments and were randomized in a 1:1 ratio to the HIICT group (n = 32) or the CT group (n = 32). All participants received the allocated intervention and completed post-intervention testing. There were no losses to follow-up or discontinuations during the 8-week intervention period, and all randomized athletes were included in the final intention-to-treat analyses (Fig 4). Attendance was 100% in both groups, with no withdrawals and no training-related injuries requiring missed sessions. For descriptive exposure screening, using the weekly group-based criterion (group mean ± 2 SD within each week and group), 21 of 512 athlete-week values (4.1%) exceeded the threshold (HIICT: 12/256; CT: 9/256). Using the individual-based criterion (each athlete’s mean ± 2 SD across the intervention), 0 of 512 athlete-week values (0%) exceeded the threshold. Collectively, these findings indicate no extreme within-athlete deviations in weekly exposure, while the small number of group-based outliers likely reflects expected between-athlete variability in perceived exertion under the same program. These observations were used solely for descriptive quality-control reporting and did not result in any data removal or imputation. All analyses were conducted on the full dataset including all 512 athlete-week values (intention-to-treat).

Across the 8-week intervention, cumulative internal load (sRPE-load, AU) was comparable between groups (HIICT: 14,001.67 AU; CT: 13,777.43 AU). Mean weekly internal load was 1,750.21 ± 79.63 AU in HIICT and 1,722.18 ± 73.96 AU in CT (Table 4).

**Table 4:** Weekly sRPE-load in the HIICT and CT groups during the 8-week intervention.

Week	HIICT (AU)	CT (AU)	Difference (HIICT−CT)
1	1685.00	1606.69	78.31
2	1773.13	1780.88	−7.75
3	1795.19	1772.38	22.81
4	1653.50	1797.16	−143.66
5	1794.78	1713.00	81.78
6	1644.91	1613.47	31.44
7	1787.66	1757.72	29.94
8	1867.50	1736.13	131.37

Table 5 and Table 6 summarize baseline and post-intervention outcomes for the HIICT and CT groups (S1 Table). For time-based variables (100 m, 200 m, first 60 m) and ground contact time, negative changes indicate faster performance (i.e., improvement).

**Table 5:** Sprint performance and neuromuscular outcomes before and after the 8-week intervention (mean ± SD).

Outcome	HIICT (Pre)	HIICT (Post)	CT (Pre)	CT (Post)	Change in HIICT, mean (95% CI)	Change in CT, mean (95% CI)	Between-group difference in change, mean (95% CI)	Group×Time p	ηp²
Weight (kg)	69.66 ± 2.36	69.41 ± 2.30	69.89 ± 2.35	70.09 ± 2.26	−0.25 (−0.56, 0.06)	0.20 (−0.12, 0.51)	−0.44 (−0.87, −0.01)	0.044	0.064
Body fat (%)	13.42 ± 0.89	12.73 ± 0.63	13.39 ± 0.93	12.76 ± 0.71	−0.69 (−0.84, −0.53)	−0.63 (−0.81, −0.45)	−0.06 (−0.29, 0.18)	0.630	0.004
100 m (s)	11.45 ± 0.25	11.21 ± 0.23	11.46 ± 0.26	11.39 ± 0.27	−0.24 (−0.26, −0.22)	−0.08 (−0.08, −0.07)	−0.16 (−0.18, −0.15)	<0.001 †	0.849
200 m (s)	23.08 ± 0.42	23.02 ± 0.48	23.13 ± 0.53	23.14 ± 0.52	−0.06 (−0.12, 0.01)	0.01 (−0.02, 0.04)	−0.07 (−0.14, 0.00)	0.063	0.055
First 60 m of 100 m (s)	7.35 ± 0.17	7.17 ± 0.17	7.38 ± 0.17	7.32 ± 0.17	−0.19 (−0.21, −0.17)	−0.06 (−0.07, −0.05)	−0.13 (−0.15, −0.11)	<0.001 †	0.677
Ground contact time (s)	0.119 ± 0.004	0.114 ± 0.006	0.122 ± 0.004	0.122 ± 0.006	−0.005 (−0.006, −0.004)	−0.000 (−0.001, 0.001)	−0.005 (−0.007, −0.003)	<0.001 †	0.366
10RM Squat (kg)	82.73 ± 8.34	82.27 ± 8.76	82.34 ± 8.77	83.12 ± 8.87	−0.47 (−1.42, 0.49)	0.78 (0.01, 1.56)	−1.25 (−2.46, −0.04)	0.042	0.065
10RM Bench Press (kg)	52.11 ± 4.02	51.95 ± 4.65	51.88 ± 4.26	52.50 ± 4.35	−0.16 (−1.15, 0.84)	0.62 (−0.35, 1.60)	−0.78 (−2.15, 0.58)	0.257	0.021
10RM Power Clean (kg)	60.08 ± 5.97	64.84 ± 6.32	59.92 ± 5.87	60.47 ± 6.24	4.77 (3.87, 5.66)	0.55 (−0.17, 1.26)	4.22 (3.09, 5.34)	<0.001	0.476
10RM Snatch (kg)	50.08 ± 5.55	50.62 ± 5.68	50.08 ± 5.63	50.31 ± 5.91	0.55 (−0.09, 1.18)	0.23 (−0.19, 0.65)	0.31 (−0.44, 1.06)	0.406	0.011
Relative 10RM Squat (kg/kg)	1.189 ± 0.129	1.187 ± 0.137	1.180 ± 0.137	1.187 ± 0.134	−0.002 (−0.018, 0.013)	0.007 (−0.004, 0.018)	−0.010 (−0.028, 0.009)	0.3	0.017
Relative 10RM Bench Press (kg/kg)	0.748 ± 0.054	0.749 ± 0.068	0.743 ± 0.061	0.750 ± 0.066	0.001 (−0.014, 0.015)	0.007 (−0.007, 0.021)	−0.006 (−0.026, 0.014)	0.525	0.007
Relative 10RM Power Clean (kg/kg)	0.864 ± 0.094	0.936 ± 0.100	0.859 ± 0.094	0.864 ± 0.096	0.072 (0.058, 0.085)	0.005 (−0.006, 0.016)	0.067 (0.049, 0.084)	<0.001	0.487
Relative 10RM Snatch (kg/kg)	0.720 ± 0.086	0.730 ± 0.085	0.718 ± 0.086	0.719 ± 0.088	0.010 (0.001, 0.019)	0.001 (−0.006, 0.008)	0.009 (−0.002, 0.020)	0.107	0.041
CMJ (cm)	53.45 ± 3.11	58.13 ± 3.23	53.43 ± 3.40	53.25 ± 3.37	4.68 (4.10, 5.26)	−0.18 (−1.12, 0.76)	4.86 (3.77, 5.94)	<0.001†	0.563
SJ (cm)	38.19 ± 3.31	40.86 ± 3.98	38.14 ± 3.17	39.32 ± 3.36	2.67 (1.82, 3.52)	1.18 (0.08, 2.28)	1.49 (0.12, 2.85)	0.033†	0.071

†: P-Value after Holm–Bonferroni correction (m = 6).

**Table 6:** Anaerobic power outcomes before and after the 8-week intervention (mean ± SD).

Outcome	HIICT (Pre)	HIICT (Post)	CT (Pre)	CT (Post)	Change in HIICT, mean (95% CI)	Change in CT, mean (95% CI)	Between-group difference in change, mean (95% CI)	Group×Time p	ηp²
Peak power (W)	559.90 ± 70.49	597.12 ± 68.89	562.15 ± 73.12	564.93 ± 74.72	37.22 (32.68, 41.75)	2.78 (−1.05, 6.61)	34.43 (28.61, 40.25)	<0.001	0.693
Relative peak power (W/kg)	8.04 ± 0.99	8.61 ± 0.98	8.05 ± 1.03	8.06 ± 1.04	0.57 (0.51, 0.62)	0.02 (−0.02, 0.05)	0.55 (0.49, 0.61)	<0.001†	0.828
30 s power decline (%)	58.64 ± 4.15	54.95 ± 3.98	58.64 ± 4.24	58.70 ± 4.43	−3.69 (−4.11, −3.27)	0.06 (−0.20, 0.32)	−3.75 (−4.23, −3.26)	<0.001	0.794

†: P-Value after Holm–Bonferroni correction (m = 6).

### Body composition and strength-related outcomes

Body mass changed only slightly during the intervention. Although the Group × Time interaction was statistically significant (p = 0.044, ηp² = 0.064), the mean changes were small in both groups, and the 95% CI for the HIICT group crossed zero. Body fat percentage decreased in both groups, with no evidence of a differential change between groups (interaction p = 0.630, ηp² = 0.004). For absolute 10RM squat, a significant Group × Time interaction was observed (p = 0.042, ηp² = 0.065). However, the direction of change showed a small decrease in HIICT and a small increase in CT (−0.47 kg vs. + 0.78 kg; between-group difference in change = −1.25 kg, 95% CI −2.46 to −0.04). Therefore, although this interaction was statistically significant, its practical relevance should be interpreted cautiously because the absolute magnitude of change was small and relative 10RM squat did not show a meaningful between-group difference in change. Similarly, 10RM snatch and relative 10RM bench press did not show meaningful between-group differences in change.

In contrast, power-clean performance showed a clearer group difference. Absolute 10RM power clean increased more in HIICT than in CT (interaction p < 0.001, ηp² = 0.476). A similar pattern was observed for relative 10RM power clean (interaction p < 0.001, ηp² = 0.487). Overall, the largest effects in this outcome domain were observed for power-clean performance, whereas body composition and most other strength-related outcomes showed only small or limited between-group differences.

### Sprint and jump outcomes

Sprint and jump outcomes generally favored HIICT. The 200-m time did not show a clear between-group difference in change (interaction p = 0.063, ηp² = 0.055). However, clear Group × Time interactions were observed for 100-m time, first-60-m split time, ground contact time, and CMJ.

For 100-m performance, HIICT showed a larger improvement than CT (−0.24 s vs. −0.08 s; interaction p < 0.001, ηp² = 0.849). A similar pattern was found for the first-60-m split (−0.19 s vs. −0.06 s; interaction p < 0.001, ηp² = 0.677). Ground contact time decreased in HIICT but remained essentially unchanged in CT, also producing a significant interaction (p < 0.001, ηp² = 0.366).

Jump outcomes followed the same general pattern. CMJ increased in HIICT but showed no meaningful change in CT (interaction p < 0.001, ηp² = 0.563). SJ improved in both groups, with a smaller but statistically significant between-group difference in change after Holm–Bonferroni correction (interaction adjusted p = 0.033, ηp² = 0.071). These findings indicate that HIICT produced more favorable adaptations in sprint-specific and jump-related outcomes, especially in measures linked to acceleration, high-speed running mechanics, and explosive lower-limb performance. The large effect sizes for 100-m time, first-60-m split, ground contact time, and CMJ indicate that the two groups followed clearly different change patterns over the intervention period.

### Anaerobic power outcomes

**Fig 5:**
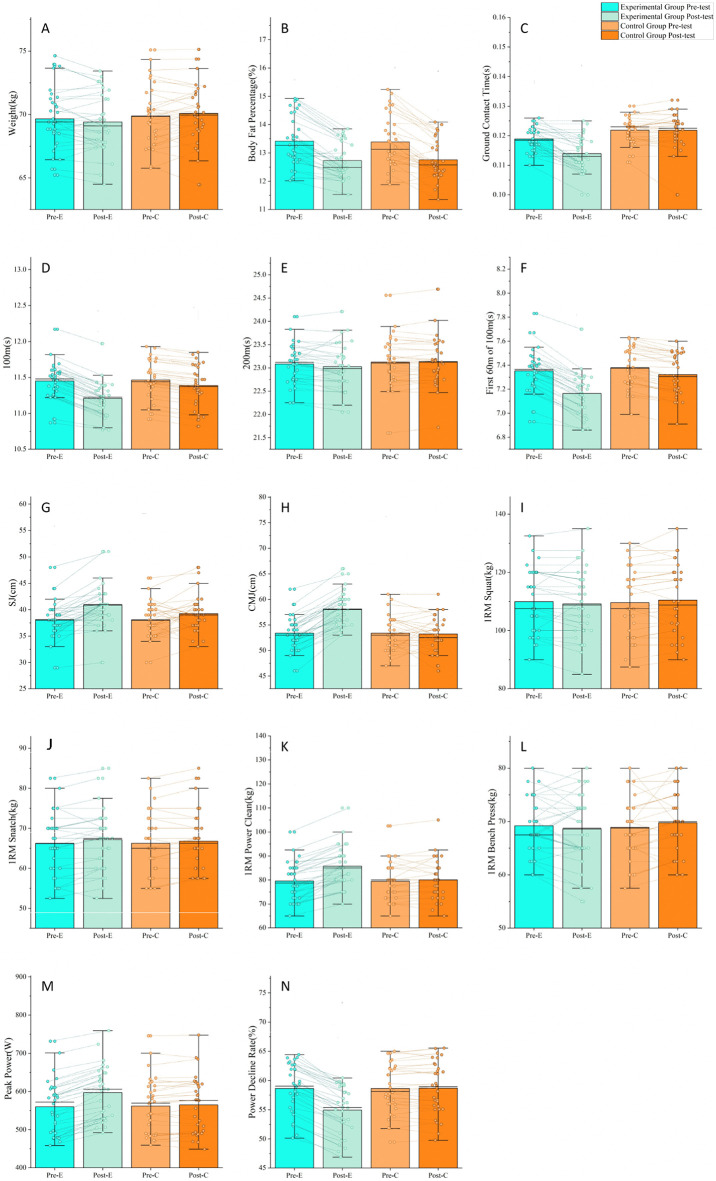
Difference in Performance of Experimental and Control Groups. (A) Body mass; (B) body fat percentage; (C) ground contact time; (D) 100-m sprint time; (E) 200-m sprint time; (F) first-60-m split time of the 100-m sprint; (G) squat jump height; (H) countermovement jump height; (I) one-repetition maximum squat; (J) one-repetition maximum snatch; (K) one-repetition maximum power clean; (L) one-repetition maximum bench press; (M) Wingate peak power; and (N) Wingate power decline rate. Bars represent the mean ± standard deviation, circles represent individual participant observations, and connecting lines link the pre- and post-intervention values of each participant. HIICT, high-intensity interval cross training; CT, classical training; SJ, squat jump; CMJ, countermovement jump; 1RM, one-repetition maximum.

Anaerobic power outcomes also showed a consistent pattern favoring HIICT (Table 6; Fig 5). Compared with CT, HIICT produced larger improvements in peak power, relative peak power, and 30-s power decline rate.

Peak power increased more in HIICT than in CT (37.22 W vs. 2.78 W; interaction p < 0.001, ηp² = 0.693). Relative peak power also improved in HIICT but remained essentially unchanged in CT (interaction p < 0.001, ηp² = 0.828). In addition, the 30-s power decline rate decreased in HIICT, whereas no meaningful change was observed in CT (interaction p < 0.001, ηp² = 0.794). These large effect sizes suggest that HIICT produced more consistent improvements in anaerobic power and fatigue-resistance-related performance than CT.

## Discussion

Across the intervention, HIICT produced larger improvements than CT in sprint and neuromuscular outcomes (Table 5). Specifically, 100 m time improved by −0.24 s (95% CI −0.26 to −0.22) in HIICT versus −0.08 s (95% CI −0.08 to −0.07) in CT (between-group difference in change −0.16 s, 95% CI −0.18 to −0.15; Group×Time p < 0.001; ηp² = 0.849). Similar patterns were observed for the first 60 m split (−0.19 vs −0.06 s; between-group difference −0.13 s, 95% CI −0.15 to −0.11; p < 0.001; ηp² = 0.677), ground contact time (−0.005 vs −0.000 s; between-group difference −0.005 s, 95% CI −0.007 to −0.003; p < 0.001; ηp² = 0.366), and CMJ height (4.68 vs −0.18 cm; between-group difference 4.86 cm, 95% CI 3.77 to 5.94; p < 0.001; ηp² = 0.563). SJ improved in both groups, but the magnitude was greater following HIICT (2.67 vs 1.18 cm; between-group difference 1.49 cm, 95% CI 0.12 to 2.85; p = 0.033; ηp² = 0.071). Consistent with these neuromuscular changes, Wingate-derived anaerobic indices also favored HIICT (Table 6), including relative peak power (0.57 vs 0.02 W·kg^-1^; between-group difference 0.55 W·kg^-1^, 95% CI 0.49 to 0.61; p < 0.001; ηp² = 0.828) and a reduction in 30 s power decline (−3.69% vs 0.06%; between-group difference −3.75%, 95% CI −4.23 to −3.26; p < 0.001; ηp² = 0.794). In contrast, body fat decreased similarly in both groups (between-group difference −0.06%, 95% CI −0.29 to 0.18; p = 0.630). Weekly sRPE-load varied across the intervention, which is expected in applied training settings. However, overall internal load was comparable between groups, suggesting that the main between-group differences were unlikely to be explained by differences in perceived training load. Instead, the findings may be more closely related to training specificity, as HIICT included more sprinting and jumping actions that matched the tested outcomes.

### Physiological mechanism

The physiological mechanisms underlying the observed improvements should be interpreted cautiously because neuromuscular activity, muscle-tendon stiffness, muscle morphology, and metabolic responses were not directly measured in this study. The improvements in CMJ, SJ, ground contact time, power-clean performance, and Wingate-derived relative peak power suggest more favorable changes in explosive lower-limb performance and repeated high-intensity power output after HIICT. These outcomes are consistent with possible improvements in rapid force production, stretch-shortening cycle function, movement coordination, and fatigue-resistance-related capacity. However, these mechanisms remain explanatory hypotheses rather than confirmed findings from the present data. Future studies should include direct biomechanical, electromyographic, and metabolic measurements to clarify how HIICT may influence sprint- and power-related performance in junior sprinters [23–27].

### Athletic performance

After eight weeks of intervention, HIICT produced more favorable changes than CT in sprint performance, jump performance, and Wingate-derived anaerobic power outcomes. These findings are consistent with previous evidence showing that high-intensity circuit training can improve multiple performance outcomes in youth athletes. For example, previous research in youth badminton players showed improvements in sport-specific strokes, repeated strength-endurance tasks, sprint time, and standing long-jump performance after high-intensity circuit training. This supports our finding that a multi-modal high-intensity program may improve both explosive and repeated-effort capacities in young athletes. Similarly, Boraczyński et al. reported that HICT improved standing long-jump and 10 × 5 m shuttle-run performance in adolescent soccer players [28]. This directly parallels our observed improvements in CMJ, SJ, and sprint-related outcomes, suggesting that high-intensity multi-joint training may transfer to lower-limb explosive and acceleration-related performance. However, because those studies involved different sports and training contexts, their findings should be used as supportive evidence rather than direct proof of the effects of HIICT in junior sprinters.

In addition, the HIICT group showed larger improvements in power-clean performance than the CT group. One possible explanation is that the sprinting and jumping components of HIICT provided more specific stimuli for rapid force production and high-velocity movement coordination. From a training-practice perspective, previous research on youth resistance training and weightlifting, traditional resistance training, and plyometric interventions suggests that these modalities can improve strength-, power-, and speed-related qualities [29,30]. These findings provide contextual support for the plausibility of transfer to power-clean performance, but they should not be interpreted as direct evidence of the mechanisms underlying the present results. Another possible explanation is that residual fatigue from strength-oriented loading near the post-test period may have influenced strength and power expression in the CT group [31]. However, this interpretation remains speculative because pre-test fatigue, recovery status, and tapering responses were not directly measured. Future studies should include fatigue and recovery markers, direct neuromuscular and biomechanical measurements, and standardized tapering strategies to clarify the mechanisms by which HIICT may influence sprint and power performance.

### Body composition

Body fat percentage decreased in both groups, whereas body mass changed only slightly. This finding is broadly consistent with Ömür et al. [32], who reported favorable changes in body mass index, body weight, and body fat percentage after an 8-week HIICT program. The reduction in body fat may be partly related to the high energy demand of interval- and circuit-based training, including increased post-exercise oxygen consumption and lipid oxidation [33]. In addition, the sprinting, plyometric, and resistance-based components of both programs may have helped preserve lean tissue through resistance-exercise-related muscle protein remodeling [34]. This may partly explain why body mass changed only slightly despite reductions in body fat. However, because body fat decreased similarly in both groups, HIICT did not show a clear advantage over CT for body-composition change. Therefore, these findings should be interpreted cautiously and should not be considered the primary advantage of HIICT in the present study [35].

### Limitation

This study has several limitations. First, participants and coaches could not be blinded to group allocation because the training protocols were clearly different. This may have introduced performance or expectation bias during the intervention. Although outcome assessors were unaware of group assignments and statistical analyses were conducted using coded group labels, the lack of participant and coach blinding should still be considered when interpreting the findings.

Second, the study design limits comparisons with other high-intensity training models. Although HIICT showed more favorable changes than CT in several outcomes, the absence of a dedicated HIIT group prevents direct comparison between HIICT and traditional HIIT.

Third, the intervention lasted only 8 weeks. Therefore, the present findings cannot determine whether the observed changes would be maintained over a longer training period or whether HIICT influences long-term outcomes such as injury risk, sustained performance development, or physiological adaptation.

Fourth, the participants were male junior sprinters from a specific training context. This limits the generalizability of the findings to female athletes, athletes from other age groups, and athletes in other sports. Future studies should include more diverse samples and longer follow-up periods.

Fifth, several measurement methods should be interpreted as field-based assessments rather than laboratory gold-standard measurements. The 10RM tests were selected for safety and feasibility in adolescent athletes, but they may be influenced by technical fatigue, especially in complex Olympic-style lifts such as the snatch and power clean [36]. Similarly, jump height and ground contact time were derived from smartphone-based video analysis. Although previous studies support the field-based validity of smartphone and Kinovea analysis, these methods are less precise than force plates or optoelectronic systems [18,37,38]. This limitation is directly relevant to the interpretation of the main findings because jump height and ground contact time were key outcomes in the present study. Therefore, although the observed changes in CMJ, SJ, and ground contact time favored HIICT, the magnitude of these changes should be interpreted with caution. Future studies should confirm these findings using laboratory-grade biomechanical instruments, such as force plates, photocell systems, wearable sensors, or three-dimensional motion-capture systems.

## Conclusion

In male junior sprinters from this specific training context, an 8-week HIICT program performed under comparable internal training loads was associated with more favorable short-term changes than CT in selected sprint-related neuromuscular outcomes, including acceleration-related performance, jump performance, ground contact time, and relative peak power. These findings suggest that HIICT may be considered as a supplementary option when the goal is to target sprint-specific explosive qualities in this population. However, because this study included only male junior sprinters and used field-based assessment methods, the findings should be interpreted cautiously. Future studies should confirm these results using more precise biomechanical measures, longer follow-up periods, and more diverse samples, including female athletes, different age groups, and athletes from different training backgrounds.

## Supporting information

S1 Table Participant-level outcome data and daily sRPE records. This table includes pre- and post-intervention outcome data and daily session rating of perceived exertion records used in the main analyses.
(XLSX)

S1 File Application Form for Ethical Review of Tianjin University of Sport. (PDF)
