# Supplementary material for: Effects of high-intensity interval cross training and classical training on physical and sprint performance in male junior sprinters
Source: PLoS One. 2026 Aug 3;21(8):e0354592. doi: 10.1371/journal.pone.0354592 (PMC13432149; doi:10.1371/journal.pone.0354592)
Supplement: S1 File — (PDF) [file pone.0354592.s002.pdf]

Application Form for Ethical Review of Tianjin University of Sport

No. **TJUS**-2025-004

<table>
<tr><td colspan="6">Project Name: Effects Between High-Intensity Interval Cross Training and Classical Training on The Male Junior Sprinters</td></tr>
<tr><td>Leader</td><td>Z.Z</td><td>title</td><td>Student</td><td>Department</td><td>School of Physical Education</td></tr>
<tr><td>Phone number</td><td>+86 15964205925</td><td>Members</td><td colspan="3">Z.Z; L.C; H.S; W.J; J.Z; W.X; X.Y</td></tr>
<tr><td>Type</td><td colspan="5">☐Project Application ☐Approved Project ☐Continuation Project
☐Commissioned Project ☑Published Paper ☐Other (Please Specify):_________</td></tr>
<tr><td colspan="6">Funding Sources: ☐Government ☐Foundation ☐Company ☐International Organization ☑Other</td></tr>
<tr><td colspan="6">Materials : ☑ Scheme ☐Informed Consent Form ☐Other:</td></tr>
<tr><td colspan="3">Does it involve human research: ☑Yes ☐No</td><td colspan="3">Does it involve animal research: ☐Yes ☑No</td></tr>
<tr><td colspan="6">Abstract:
This study focuses on young male sprinters and systematically examines the effects of an 8-week HIITC intervention on body composition, specialized performance, general physical fitness, and physiological indicators. The study recruited 64 adolescent athletes from a track and field club and randomly assigned them to either an experimental group or a control group, with 32 participants in each.
The experimental group underwent an 8-week HIITC training program, while the control group continued with their conventional training regimen. Tests were conducted at both the beginning and the end of the training period. The test contents and methods followed the guidelines for adolescent physical training issued by the American Physical Fitness Association, ensuring no adverse effects on their growth and development.
Both groups followed a scientifically designed training regimen, with intensity and duration carefully controlled to align with the participants' physical capacity. The HIITC training regimen implemented in the experimental group, designed by the research team, adhered to standard physiological norms, with no abnormal changes observed in physiological indicators.</td></tr>
<tr><td colspan="6">Review Category: ☑ Accelerated Review ☐ Meeting Review</td></tr>
<tr><td colspan="6">Conclusion: ☑ Agree ☐ Agree After Necessary Modification ☐ Disagree ☐ Suspend Or Terminate The Test</td></tr>
<tr><td colspan="6">The applicant (Project Leader) promises:
All the above contents are true. If approved, I will study in strict accordance with the scheme provided and comply with the relevant regulations of the ethics committee of Tianjin Institute of physical education.
Sign:
Date: 2025/1/25</td></tr>
<tr><td colspan="6">Review comments of the ethics committee:
1. the researcher is qualified to carry out the study.
2. the research protocol and informed consent basically meet the ethical requirements. Please follow the relevant laws, regulations and rules of China, and follow the scheme and informed consent approved by the ethics committee of Tianjin Institute of physical education.
Ethics Committee of Tianjin University of Sport (seal)
Approval date: 2025/1/26</td></tr>
</table>

Note: this form is in triplicate, and the applicant, the ethics committee and the scientific research department keep one copy each.

天津体育学院伦理审查申请表

伦理编号：TJUS-2025-004

项目名称： Effects Between High-Intensity Interval Cross Training and Classical Training on The Male Junior Sprinters

**项目负责人**	张镇宇	**职称**	学生	**单位/部门**	体育教育学院
**联系电话**	15964205925	**课题组成员**	张镇宇、刘承豪、郝诗语、王娇洁、靳泽宇、王馨迤、谢云		
**申请审查类型**	☐申请项目 ☐批准后项目 ☐延续项目 ☐委托项目 ☑发表论文<br>☐其他（请注明）：				

研究项目经费来源： ☐政府 ☐基金会 ☐公司 ☐国际组织 ☑其他

递交审查资料： ☑方案 ☐知情同意书 ☐其他资料：

是否涉及人体研究： ☑是 ☐否　　**是否涉及动物研究：** ☐是 ☑否

研究内容及研究方案摘要：

本研究以青少年男子短跑运动员为对象，通过为期 8 周的 HIICT 干预，系统考察其在身体形态、专项成绩、一般体能及生理学指标等方面的影响。

实验选取某田径俱乐部 64 名青少年作为研究对象，其中随机分为实验组和对照组各 32 人，实验组接受为期 8 周的 HIICT 训练，对照组维持常规训练模式。在训练开始与结束时，分别进行一次测试，测试内容与方法均采用美国体能协会体能训练指导中，对青少年的测试方法，确保对青少年生长发育不产生负面影响。

两组均采用合理的训练模式，训练强度与时间控制在青少年能承受的范围之内，实验组采取的 HIICT 训练模式，经课题组成员以自身为受试者确定，对人体的符合施加在正常的范围，不产生生理指标的异常变化。

审查类别： ☑ 加快审查　☐ 会议审查

结论： ☑同意　☐作必要修改后同意　☐不同意　☐暂停或终止试验

申请人（项目负责人）承诺：

以上所填内容均属实，如获批准，我将严格按照提供的方案进行研究，并遵守天津体育学院伦理委员会的相关规定。

申请人（项目负责人）签字：[illegible]

日期：2025 年 1 月 25 日

伦理委员会审查意见：

经本伦理委员会审查：

1.研究者具备开展该研究的资格。

2.研究方案及知情同意书基本符合伦理要求。

请遵循我国相关法律、法规和规章，并遵循天津体育学院伦理委员会批准的方案和知情同意书开展研究。

天津体育学院伦理委员会（盖章）

批准日期 2025 年 1 月 26 日

备注： 此表一式 3 份，申请人、伦理委员会及科研处各存 1 份。
